# Early prevention and risk factors analysis of portal vein system thrombosis after laparoscopic splenectomy and pericardial devascularization

**DOI:** 10.1007/s00464-022-09340-5

**Published:** 2022-06-28

**Authors:** Yongning Li, Xueqin Fu, Ying Li, Peng Liu, Songbai Liu, Yaozhen Pan

**Affiliations:** 1 Department of Hepatobiliary Surgery, The Affiliated Hospital of Guizhou Medical University, Guiyang, Guizhou China; 2 College of Clinical Medicine, Guizhou Medical University, Guiyang, Guizhou China; 3 Department of Breast Surgery, Guizhou Provincial People’s Hospital, Guiyang, Guizhou China; 4 Department of Hepatobiliary Surgery, The Affiliated Cancer Hospital of Guizhou Medical University, Guiyang, Guizhou China

**Keywords:** Warfarin, Liver cirrhosis, Portal hypertension, Portal vein thrombosis, Splenectomy

## Abstract

**Background:**

Portal vein system thrombosis (PVST) is a common postoperative complication brought by laparoscopic splenectomy and pericardial disconnection (LSD) among patients who suffered from portal hypertension and hypersplenism. This research lies mainly in probing into the risk factors of PVST and evaluating the effects of warfarin on PVST prevention.

**Materials and methods:**

We took 131 individuals who have carried out LSD from January 2015 to January 2021. Patients were divided into warfarin group (*n* = 68) and aspirin group (*n* = 63). Meanwhile, thrombosis factors were analyzed in PVST arm (*n* = 48) and non-PVST arm (*n* = 83).

**Results:**

We analyzed the early postoperative anticoagulation effect, 20 patients (29.4%) in the warfarin group developed PVST, and 28 patients (44.4%) in the aspirin group. The chance to PVST during the first year after operation was lower in the warfarin group than in the aspirin group (*F* = 13.43, *P* = 0.006). Risk factors for PVST were analyzed, and diabetes, the diameter of the portal vein and splenic vein, and the velocity of portal blood flow were statistically significant between the PVST arm and non-PVST arm (*P* <  < 0.05). Multiple logistic regression analyses have shown that diabetes, portal vein diameter, splenic vein diameter, and the velocity of portal blood flow were the risk factors of PVST.

**Conclusions:**

The portal vein diameter, splenic vein diameter, portal vein flow velocity, and diabetes are risk factors for the PVST after LSD. The prophylactic use of warfarin anticoagulation markedly decreases the probability of occurrence of the PVST in patients with portal hypertension after LSD compared to aspirin.

**Graphical abstract:**

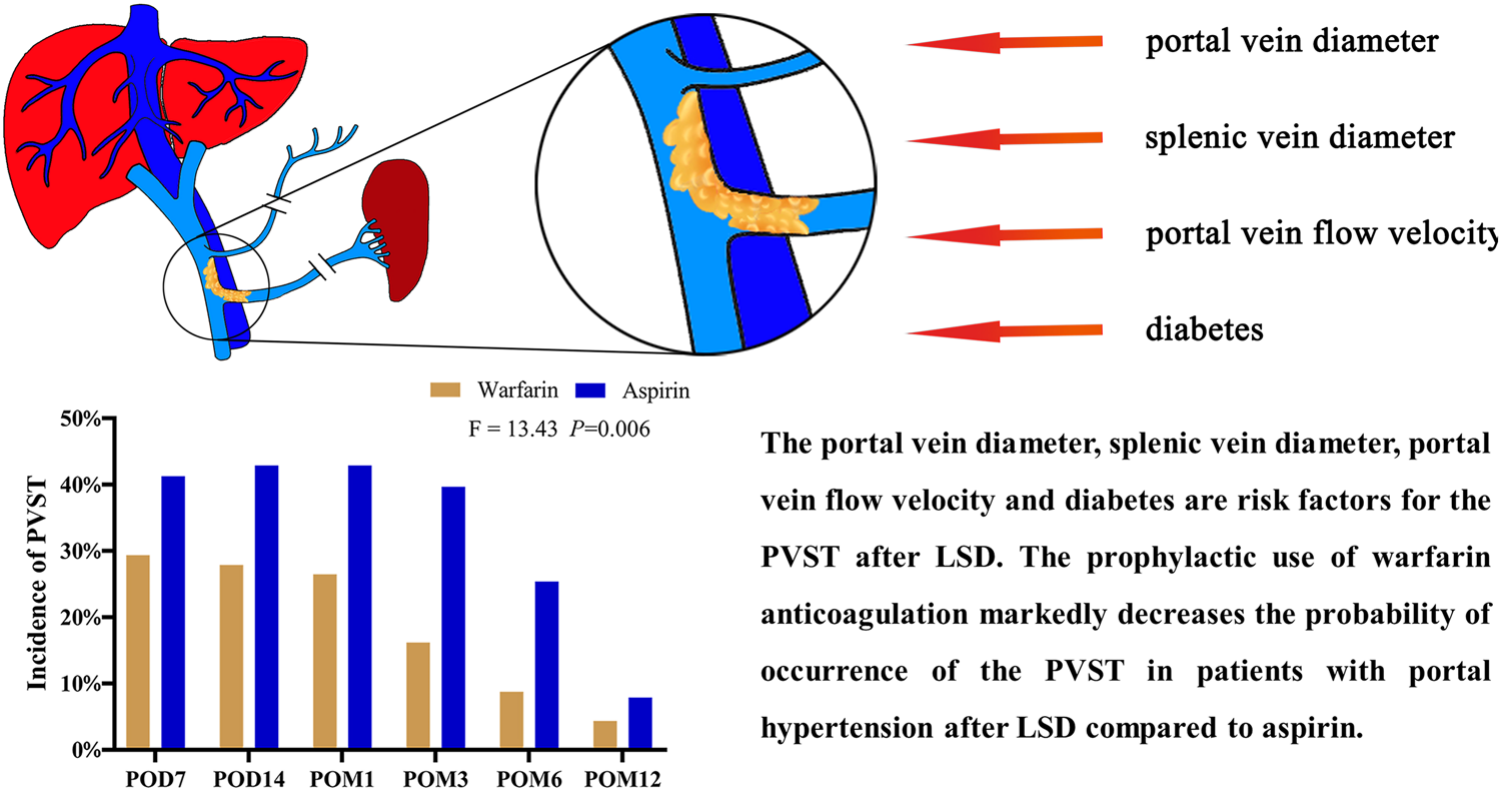

**Supplementary Information:**

The online version contains supplementary material available at 10.1007/s00464-022-09340-5.

Liver cirrhosis is a frequently occurring chronic liver disease, which is usually asymptomatic in early stage and portal hypertension in later stage [1]. In patients with portal hypertension, hypersplenism and upper gastrointestinal bleeding are serious complications [2]. Nowadays, because of advances in endoscopic technology, laparoscopic splenectomy and pericardial devascularization (LSD) is the main method for the treatment of hypersplenism and upper gastrointestinal bleeding [3]. It has been widely reported that patients after LSD surgery are prone to portal vein system thrombosis (PVST) [3, 4], but reports on the incidence rate of PVST in different centers are inconsistent, about 18.9–57% [5–7]. However, there are only a few reports of PVST after laparoscopic splenectomy in spleen rupture or hematologic diseases, which could be linked to the pathophysiological features of portal hypertension [8].

PVST means a thrombosis occurring in the splenic vein, portal vein, superior mesenteric vena, or intrahepatic portal vein, the most common of which is the thrombosis occurring in the splenic vein [9]. PVST can exacerbate portal hypertension, resulting in refractory ascites, hepatic encephalopathy, and esophagogastric variceal bleeding [10]. So far, the specific mechanism of PVST formation is not completely clear, but some studies have suggested that it may be related to the surgical method, coagulation mechanism, hemodynamic changes, and perioperative intervention [11, 12]. Due to the differences in the degree, location, and onset time of PVST, its clinical manifestations are often atypical and its therapeutic is not effective. Although LSD has been widely carried out in many centers, there are still few reports on postoperative complications of LSD, and there are controversies about the factors leading to PVST in patients after LSD. Therefore, it is particularly important to find its formation factors and conduct a comparative analysis.

Despite the fact that many studies have shown that routine prophylactic anticoagulant therapy after splenectomy can reduce the risk of PVST formation, it is unclear which anticoagulants are effective in preventing PVST after LSD. It has been suggested that common antiplatelet drugs like aspirin and clopidogrel are ineffective in preventing PVST [13]. However, warfarin, the most commonly used oral anticoagulant in clinical practice, is relatively inexpensive but rarely reported to prevent PVST.

Hence, the current research targeted to argue the major factors for PVST and evaluate the effectiveness of warfarin in preventing PVST formation following LSD. From January 2015 to January 2021, we compared the effectiveness of warfarin and aspirin in the postoperative prophylaxis of PVST in patients with portal hypertension who underwent LSD surgery at our center.

## Materials and methods

### Participants

All individuals with cirrhosis hypersplenism admitted to the Department of Hepatobiliary Surgery, Affiliated Hospital of Guizhou Medical University, and The Affiliated Cancer Hospital of Guizhou Medical University from January 2015 to January 2021 were recruited. The admission criteria were as follows: The research object was aged from 18 to 80 years and had been diagnosed with portal hypertension and hypersplenism; History of esophagogastric variceal bleeding and Childe-Pugh A or B liver function did not have PVST, and detected by CT, MRI, or Doppler ultrasound before operation; and successfully underwent LSD.

The exclusion criteria were as follows: The patient was diagnosed with liver cancer or any other malignant tumor; there are coagulopathy or hypercoagulable unrelated to liver disease; there is evidence of clot within the portal vein system prior to undergoing the LSD procedure; combined with serious angiocardiopathy, lung, kidney dysfunction or blood system disease; baseline international normalized ratio (INR) > 2; pregnancy; previous history of the cardiovascular and cerebrovascular hemorrhagic disease: infected with immunodeficiency virus.

### Study design

In this study, patients in both groups were subcutaneously injected with low-molecular weight heparin (ALFA WASSERMANN, Bologna, Italy) (4250 U/d) for 5 days and orally dipyridamole (CSPC, Shijiazhuang, China) 25 mg 3 times a day for 3 months from the POD1. From postoperative day (POD) 3, the experimental group was given warfarin sodium (QILU, Shandong, China) orally for 6 months. The pre-dose was 2.5 mg/d, and the dose was quickly adjusted to achieve the target INR of 2–3. The control group was given enteric-coated aspirin tablets (Bayer, Leverkusen, Germany) orally at 100 mg/d on the POD3 for 6 months. Warfarin or aspirin should not be used if PVST does not occur within 6 months after surgery. All the patients have completed clinical observation during the 18 months of follow-up.

Once PVST was confirmed after LSD, the patients would receive a thrombolytic therapy. Urokinase was administered via the peripheral vein with a bonus dose of 250,000 units within 10 min, followed by a continuous infusion of 5000 units/h for 48 ~ 72 h via a micro-infusion pump. Following the thrombolytic treatment, the patients were administrated with oral warfarin or aspirin plus dipyridamole according to the different groups. The drug doses were adjusted based on the INR and PLT levels.

Preoperative information was retrospectively collected, including gender, age, body mass index (BMI), diabetes, Childe-Pugh classification, leukocyte (WBC) count, platelet count (PLT), total bilirubin (TBIL), alanine transaminase (ALT), blood urea nitrogen (BUN), creatinine (CRE), INR, fibrinogen (FIB), spleen longitudinal diameter, portal vein diameter, splenic vein diameter, and speed of portal blood flow. Intraoperative messages included duration of operation and estimated intraoperative blood loss. Postoperative materials included WBC, PLT, TBIL, ALT, BUN, CRE, INR, FIB, and postoperative anticoagulant treatment regimen.

Participants were randomly assigned to warfarin group and aspirin group (warfarin group odd and aspirin group even) according to the order in which they were enrolled. Patients were treated with warfarin or aspirin plus dipyridamole according to the different groups. Ultrasound and radiologists were blinded to the randomization results. The present study had approval from the Human Research Ethics Committee at the Affiliated Hospital of Guizhou Medical University. All sufferers were informed and consented.

### Operative technique

The patients were placed in supine position. The operation was performed under general anesthesia. A laparoscopic trocar was inserted into the umbilicus through a 12 mm incision, and the pneumoperitoneum was set to 9 ~ 10 mmHg. Following the successful establishment of the pneumoperitoneum, three additional trocars were inserted at the level of the subxiphoid, the left subcostal margin midclavicular line, and the left flank level. First, pericardial devascularization was using a LigaSure vessel-sealing instrument (Covidien, Norwalk, CT, USA). Then splenogastric ligaments, splenocolic ligament, and lienorenal ligament were divided by LigaSure. In the tail of the pancreas, the splenopancreatic fascia was cut and identified. With an endoscopic linear stapler, splenic hilar was performed. The spleen was removed from the umbilical incision, placed in a plastic bag, and removed.

### The detection and diagnosis of PVST

PVST formation refers to venous thromboembolic events of the splenic vein, the main branch of the portal vein, and the intrahepatic branch vein. The primary endpoint of this work was PVST. The patients routinely underwent enhanced CT 3 days before the operation. Ultrasound examinations were performed at 1, 2, 4, and 12 weeks after surgery, as well as every three months after that. However, due to the influence of the operator's tech and the patient's body habitus, thrombus may not be detected by ultrasound scanning. Therefore, a contrast-enhanced CT scan is to confirm the diagnosis if the ultrasound reveals suspicious echogenic material or reduced or no blood flow in the portal venous system lumen [14]. The location of the PVST and its extension in the portal vein main and/or intrahepatic branches and splenic vein were defined. Review medical records are retrospectively to document any other medical conditions.

### Statistical analysis

Files are depicted as mean (standard deviation), median (interquartile range), or percentage. Dual-tailed non-paired Student’s t-test was applied for structural comparison. After the chi-square test, the classified data were expressed as frequencies. The factors of PVST occurrence were discussed by single-variable analysis, and then meaningless variables were gradually removed while the logistic regression model for used in multivariate analysis. A *P* value < 0.05 was defined as having statistical significance. The statistical package for the Social Sciences (SPSS) Version 16 software (SPSS Inc., Chicago, IL, USA) works for statistical analysis.

## Results

From January 2015 to January 2021, all 156 individuals with portal hypertension complicated and secondary hypersplenism received LSD in our center. Twenty patients were excluded based on enrollment conditions in this study. The other 136 patients were randomly allocated to the warfarin group (*n* = 69) or aspirin group (*n* = 67). One patient stopped warfarin on POD5 due to bloody stools; four patients stopped aspirin treatment on POD7 due to gastrointestinal reactions. Finally, 131 patients completed treatment, including the warfarin group (*n* = 68) and aspirin group (*n* = 63).

There was no substantial difference emerged in the warfarin group and the aspirin ones, including gender, age, BMI, diabetes, Child–Pugh classification (A/B), WBC, PLT, TBIL, ALT, BUN, CRE, INR, FIB, spleen longitudinal diameter, portal vein diameter, splenic vein diameter, speed of portal blood flow, duration of surgery, and intraoperative bleeding (*P* > 0.05; Table 1).

**Table 1:** The demographics, preoperative laboratory indexes, and intraoperative clinical characteristics of warfarin and aspirin groups

Variable	Warfarin (*n* = 68)	Aspirin (*n* = 63)	*P*
Gender (female/male)	48/20	46/17	0.455
Age(years)	45.40 ± 8.19	46.59 ± 8.51	0.416
BMI	22.36 ± 2.12	22.71 ± 1.84	0.321
Diabetes	8(11.8%)	13(20.6%)	0.126
Child–Pugh (A/B)	28/40	26/37	0.566
WBC (× 10^9^/L)	2.85 ± 0.87	2.68 ± 0.76	0.291
PLT (× 10^9^/L)	44.06 ± 13.90	44.37 ± 16.59	0.787
TBIL (umol/L)	20.07 ± 9.08	18.78 ± 7.99	0.391
ALT (U/L)	32.94 ± 18.73	32.40 ± 14.12	0.758
BUN (mmol/L)	5.49 ± 1.61	4.96 ± 1.83	0.156
CRE (umol/L)	77.58 ± 22.34	79.81 ± 18.96	0.436
INR	1.34 ± 0.16	1.31 ± 0.15	0.253
FIB (g/L)	2.84 ± 0.99	2.75 ± 0.97	0.716
Longitudinal diameter of spleen (mm)	182.17 ± 30.13	185.82 ± 23.82	0.445
Portal vein diameter (mm)	13.23 ± 1.37	13.43 ± 1.73	0.478
splenic vein diameter (mm)	10.93 ± 1.75	10.81 ± 1.76	0.707
Mean portal flow velocity (cm/s)	14.58 ± 1.55	14.34 ± 1.74	0.406
Operation time (min)	129.26 ± 37.59	132.21 ± 34.24	0.460
Intraoperative blood loss (ml)	167.72 ± 112.20	175.98 ± 109.70	0.446

Data shown as mean ± standard deviation, or number of patients, as indicated

*BMI* Body Mass Index, *WBC* white blood cell, *PLT* platelets, *TBIL* total bilirubin, *ALT* alanine transaminase, *BUN* blood urea nitrogen, *CRE* creatinine, *FIB* fibrinogen

During the warfarin treatment process, INR was maintained between 2 and 3. Patients were required to take warfarin tablets after meals, the oral dose ranged from 2.0 to 7.0 mg, and the mean dose was 3.5 mg. They took daily warfarin 3.471 ± 0.373 mg at POD7, 3.713 ± 0.357 mg at POD14, 3.889 ± 0.498 mg at POM1, 3.698 ± 0.345 mg at POM3, and 3.786 ± 0.324 mg at POM6.

**Fig. 1:**
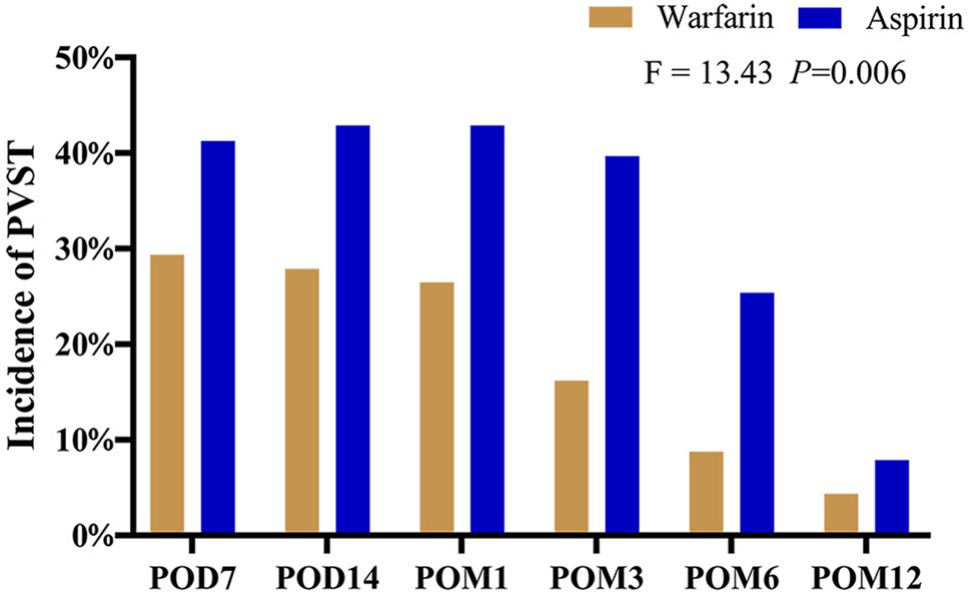
The postoperative chance of PVST in patients with portal hypertension and hypersplenism undergoing LSD

Compared with the aspirin ones, the postoperative chance of PVST in the first year was substantially lower in the warfarin group (*F* = 13.43, *P* = 0.006; Fig. 1). It was no significant difference in PVST formation frequency between the two teams at POD7 and POD14. However, at POM1, POM3, and POM6, the incidences of PVST in the warfarin group were fairly lower in contrast with the aspirin group (*P* < 0.05; Table 2).

**Table 2:** The comparison of thrombosis incidence between warfarin and aspirin groups

Time point	Warfarin (*n* = 68)	Aspirin (*n* = 63)	*P*
PVST			
POD7	20(29.4%)	26(41.3%)	0.108
POD14	19(27.9%)	27(42.9%)	0.054
POM1	18(26.5%)	27(42.9%)	0.037
POM3	11(16.2%)	25(39.7%)	0.002
POM6	6(8.8%)	16(25.4%)	0.010
POM12	3(4.4%)	5(7.9%)	0.317
MPVT			
POD7	11 (16.2%)	13 (20.6%)	0.332
POD14	10 (14.7%)	14 (22.2%)	0.188
POM1	10 (14.7%)	14 (22.2%)	0.188
POM3	6 (8.8%)	13 (20.6%)	0.047
POM6	2 (2.9%)	10 (15.9%)	0.011
POM12	1 (1.5%)	3 (4.8%)	0.281
SVT			
POD7	14 (20.6%)	18 (28.6%)	0.195
POD14	14 (20.6%)	19 (30.2%)	0.145
POM1	13 (19.1%)	19 (30.2%)	0.103
POM3	9 (13.2%)	17 (30.0%)	0.040
POM6	5 (7.4%)	10 (15.9%)	0.104
POM12	2 (2.9%)	3 (4.8%)	0.464

Data shown as number of patients (%), as indicated

*MPVT* main portal vein thrombosis, *SVT* splenic vein thrombosis

**Fig. 2:**
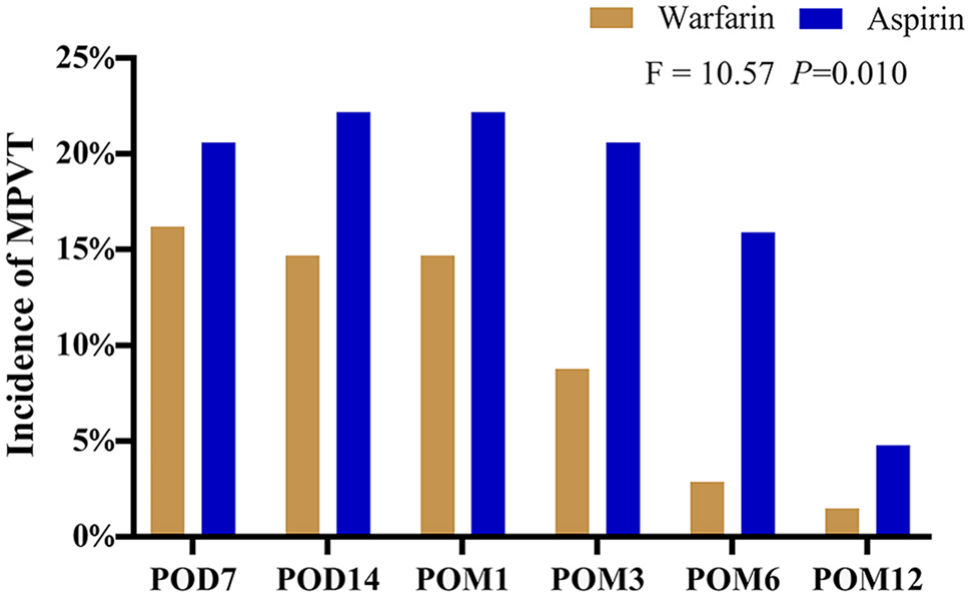
The postoperative chance of MPVT in patients with portal hypertension and hypersplenism undergoing LSD

**Fig. 3:**
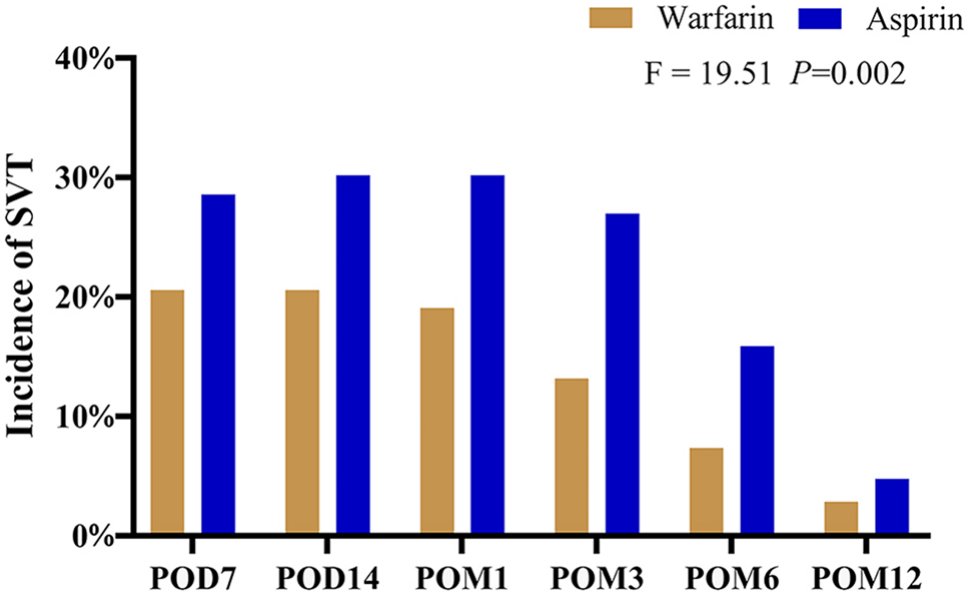
The postoperative chance of SVT in patients with portal hypertension and hypersplenism undergoing LSD

The two groups were compared for the incidence of main portal vein thrombosis and splenic vein thrombosis. In contrast with the aspirin group, the incidences of main portal vein thrombosis (MPVT) in the first year after operations were substantially lower in the warfarin team (*F* = 10.57, *P* = 0.010; Fig. 2). It was no dramatic difference in MPVT incidence among the two teams at POD 7, POD 14, POM 1, and POM 12, whereas, in POM 3 and POM 6, the MPVT incidences were dramatically fewer in warfarin than in another team (*P* < 0.05; Table 2). In contrast, the postoperative chance of splenic vein thrombosis (SVT) formed in the first year was considerably lower in warfarin than in those (*F* = 19.51, *P* = 0.002; Fig. 3). It has presented significant differences in SVT that happened at POM 3 on the two arms (*P* < 0.05; Table 2).

Then we analyzed the clinical baseline traits of the PVST and non-PVST arms in Table 3. It has not had a significant difference between the two teams, covering gender, age, BMI, Child–Pugh classification (A/B), WBC, PLT, TBIL, ALT, INR, PT, FIB, BUN, CER, spleen longitudinal diameter, duration of surgery time, and intraoperative blood loss (*P* > 0.05).

**Table 3:** The demographics, preoperative laboratory indexes, and intraoperative clinical characteristics of non-PVST and PVST groups

Variable	Non-PVST (*n* = 83)	PVST (*n* = 48)	*P*
Gender (female/male)	59/24	35/13	0.822
Age(years)	46.00 ± 8.43	45.92 ± 8.26	0.956
BMI	22.76 ± 1.93	22.13 ± 2.06	0.084
Diabetes	9(10.8%)	12(25%)	0.033
Child–Pugh (A/B)	32/51	22/26	0.415
WBC (× 10^9^/L)	2.77 ± 0.77	2.76 ± 0.91	0.697
PLT (× 10^9^/L)	43.60 ± 13.97	45.25 ± 17.21	0.871
TBIL (umol/L)	19.81 ± 8.29	18.83 ± 9.09	0.528
ALT (U/L)	31.91 ± 16.14	34.02 ± 17.49	0.390
BUN (mmol/L)	5.33 ± 1.79	5.07 ± 1.62	0.166
CRE (umol/L)	79.25 ± 21.66	77.62 ± 19.20	0.873
INR	1.31 ± 0.17	1.35 ± 0.13	0.189
FIB (g/L)	2.78 ± 0.98	2.83 ± 1.00	0.754
Longitudinal diameter of spleen (mm)	186.30 ± 27.72	179.83 ± 26.16	0.191
Portal vein diameter (mm)	13.02 ± 1.49	13.86 ± 1.52	0.002
splenic vein diameter (mm)	10.46 ± 1.67	11.59 ± 1.65	< 0.001
Mean portal flow velocity (cm/s)	14.85 ± 1.33	13.82 ± 1.91	0.001
Operation time (min)	129.93 ± 37.19	131.98 ± 33.92	0.560
Intraoperative blood loss (ml)	180.14 ± 112.62	157.08 ± 106.74	0.104

Data shown as mean ± standard deviation, or number of patients, as indicated

*BMI* Body Mass Index, *WBC* white blood cell, *PLT* platelets, *TBIL* total bilirubin, *ALT* alanine transaminase, *BUN* blood urea nitrogen, *CRE* creatinine, *FIB* fibrinogen

By univariate analysis, we confirmed that diabetes, portal vein diameter, splenic vein diameter, and the velocity of portal blood flow were statistically significant among the research teams (*P* < 0.05; Table 3). Compared to the non-PVST arm, the PVST arm had a broader portal vein diameter (*P* < 0.05), a broader splenic vein diameter (*P* < 0.05), and a slower speed of portal blood flowing (*P* < 0.001). Files have been performed by logistic multivariable regression, and certain statistically significant variables covered portal vein diameter, splenic vein diameter, and speed of portal blood flowing (Table 4).

**Table 4:** Outcome of logistic multivariate regression

Independent variables	*β*	SE	Wald	df	*P*	OR	95.0% CI
Diabetes	1.024	0.570	3.225	1	0.073	2.783	0.910–8.508
Portal vein diameter (mm)	0.396	0.159	6.205	1	0.013	1.486	1.088–2.030
splenic vein diameter (mm)	0.283	0.141	4.006	1	0.045	1.327	1.005–1.749
Mean portal flow velocity (cm/s)	− 0.413	0.138	8.952	1	0.003	0.662	0.505–0.867

*CI* confidence interval

## Discussion

PVST is one of the familiar complications accompanied by liver cirrhosis in patients suffering from portal hypertension. Based on portal vein hypertension, surgical intervention further promoted the PVST [15]. The reported incidence of PVST after LSD varies greatly, the inconsistence is possibly due to several reasons [3, 16, 17]. This may be because most patients with PVST are asymptomatic, and there are differences in examination methods, time, and frequency of postoperative examinations [12]. Our current study also showed a high incidence of PSVT after LSD. In portal hypertension or cirrhosis; however, no anticoagulant therapy is consistently recommended to prevent PVST after LSD. Therefore, we need to find the risk factors of PVST after LSD through clinical practice and use safe and effective anticoagulants to prevent PVST.

Even if the detailed mechanisms generating the PVST in post-splenectomy were still controversial, it is generally agreed that patient’s factors [18], hemorheology changes [19], coagulation mechanism [20], and irrational use of anticoagulants [21] are all associated with risk of thrombosis. In this project, the risk factors for PVST mainly included diabetes, portal vein diameter, splenic vein diameter, and portal vein flow velocity. The consequences are by to past reports [22]. Studies have confirmed that diabetic patients with hyperglycemia and insulin resistance will lead to changes in platelet count and activation, as well as qualitative and/or quantitative modifications of coagulation and fibrinolytic factors, thus, making diabetic patients more prone to thrombosis. Previous research has revealed that after splenectomy, the reduced portal vein blood flow and the residual blind end of the splenic vein caused blood stasis and turbulence which promoted the PVST [17]. Furthermore, severe SVT may develop into MPVT and mesenteric vein thrombosis which may eventually lead to intestinal blood stasis, intestinal wall edema, and ischemic necrosis [12, 16]. Broe et al. found in their study that PVST originated from the splenic vein and gradually spread into the portal vein and vena mesenterica superior [23]. The cause of the splenic vein thrombosis group is uncertain but can be explained as follows: (1) CO2 pneumoperitoneum may cause hypercoagulability in the patient's blood [24]; (2) mechanical injury of splenic vein vessels with an endoscopic cutting closure device results in exposure of subintimal collagen that activates the clotting system; and (3) Ultrasonic scalpel and Ligasure may cause thermal or oscillatory injury of venous intima [8]. As a result of the splenic vein's larger diameter, when the splenic vein is cut, the splenic vein blood flow is rapidly reduced, which may promote PVST formation [25].

In patients with liver cirrhosis complicated with portal hypertension, the total number of platelets decreases, mainly due to the increase of platelet reserve and slowing down of platelet velocity during splenomegaly, allowing enough time for them to contact splenic macrophages [9]. As a result, the spleen loses its ability to clear platelets after LSD, and the number of platelets circulating in the blood increases 2–6 times in a short period of time. According to some studies, an increase in postoperative platelet count is an important risk factor for PVST in patients with portal hypertension, and the use of anticoagulant drugs can be based on the change in postoperative platelet count [9, 25]. However, in this study, we found that although platelets rose rapidly in some patients after surgery, no PVST was formed (Table S1-2). The effects confirmed that there were no significant differences in platelet count at admission and different postoperative periods between the PVST group and non-PVST group (all *P* > 0.05), suggesting that platelets cannot be a separate pathogenic factor for PVST formation when LSD has been done. This result may be related to prophylactic anticoagulant therapy after the operation.

The diameter of the portal vein has been mentioned several times as a potentially dangerous factor in PVST formation [26]. This is consistent with the results of this study, the wider the diameter of portal vein and splenic vein of patients before surgery, the greater the decrease of portal vein pressure and the slower the blood flow velocity after surgery, and the greater the possibility of PVST after surgery.

At present, prophylactic anticoagulant therapy for PVSD after LSD has been in a dilemma for a long time, because the portal vein system may still have thrombosis in the presence of decreased platelet count and increased INR. The most concern was that early postoperative use of anticoagulants in patients with cirrhosis may increase the risk of postoperative bleeding due to the inner blood coagulation disorder. Therefore, the prophylactic anticoagulant therapy for PVST after LSD is still controversial, mainly based on personal experience. However, recent studies have proved that prophylactic anticoagulant therapy is safe and feasible, which can effectively reduce the occurrence of PVST and reduce the risk of bleeding [21, 27]. There are few randomized controlled studies on the effects of warfarin and aspirin on the prevention of postoperative PVST formation in laparoscopic splenectomy and Porto-azygos disconnection combined with portal hypertension.

Based on our 1-year randomized controlled study involving patients with cirrhosis after LSD, prophylactic use of warfarin has been proven to be effective and safe. Warfarin treatment can significantly reduce the happenchance of PVST following surgery, in contrast with aspirin treatment. In this study, we realized that the chance of PVST in warfarin teams occurred fewer than the aspirin ones at all-time points from POD7 to POM12, and the difference between the two groups from POM1 to POM6 was statistically significant. Finally, compared to aspirin, earlier postoperative use of warfarin significantly reduced the occurrence rate of PVST.

The work implemented as a single-center-based cohort study enlisted a comparatively small sample capacity, so the consequences had potential limitations. Our study subjects were cirrhotic patients with portal hypertension in Guizhou, China, and the results may have limited external validity in different regions and Settings. In addition, it is difficult for some patients to achieve the target value of 2–3 during oral warfarin adjustment of INR, which brings potential difficulties for clinical application.

## Conclusions

In conclusion, PVST is familiar postoperative comorbidity of LSD; furthermore, its incidence might be reduced by early postoperative anticoagulation. The anticoagulation effect of warfarin is better than that of aspirin. The portal vein diameter, splenic vein diameter, and portal vein flow velocity are predictors of PVST occurrence. Close postoperative follow-up and early anticoagulation are critical for patients who are at high risk of venous thrombosis formation.

## Supplementary Information

Below is the link to the electronic supplementary material.

Supplementary file1 (DOCX 18 kb)

Supplementary file2 (DOCX 18 kb)

## References

[1] Garcia-Tsao G, Abraldes JG, Berzigotti A, Bosch J (2017). Portal hypertensive bleeding in cirrhosis: risk stratification, diagnosis, and management: 2016 practice guidance by the American association for the study of liver diseases. Hepatology.

[2] Fouad TR, Abdelsameea E, Abdel-Razek W, Attia A, Mohamed A, Metwally K (2019). Upper gastrointestinal bleeding in Egyptian patients with cirrhosis: post-therapeutic outcome and prognostic indicators. J Gastroenterol Hepatol.

[3] Jiang G, Bai D, Chen P, Xia BL, Qian JJ, Jin SJ (2016). Predictors of portal vein system thrombosis after laparoscopic splenectomy and azygoportal disconnection: a retrospective cohort study of 75 consecutive patients with 3-months follow-up. Int J Surg.

[4] Zhang N, Yao Y, Xue W, Wu S (2016). Early prophylactic anticoagulation for portal vein system thrombosis after splenectomy: a systematic review and meta-analysis. Biomed Rep.

[5] Yang M, Liu J (2020). Low-molecular weight heparin prevents portal vein system thrombosis after splenectomy: a systematic review and meta-analysis. ANZ J Surg.

[6] Ruiz-Tovar J, Priego P (2017). Portal vein thrombosis after splenic and pancreatic surgery. Adv Exp Med Biol.

[7] He S, He F (2015). Predictive model of portal venous system thrombosis in cirrhotic portal hypertensive patients after splenectomy. Int J Clin Exp Med.

[8] Zhe C, Jian-wei L, Jian C, Yu-dong F, Ping B, Shu-guang W (2013). Laparoscopic versus open splenectomy and esophagogastric devascularization for bleeding varices or severe hypersplenism: a comparative study. J Gastrointest Surg.

[9] Yao W, Feng Y, Liu T, Li W, Zhang M, Yao Y (2021). Rivaroxaban versus low-molecular weight heparin plus warfarin prevents portal vein system thrombosis after splenectomy and pericardial devascularization: a randomized clinical trial. Excli j.

[10] Garcia-Pagan JC, Valla DC (2009). Portal vein thrombosis: a predictable milestone in cirrhosis?. J Hepatol.

[11] Kawanaka H, Akahoshi T, Itoh S, Iguchi T, Harimoto N, Uchiyama H (2014). Optimizing risk stratification in portal vein thrombosis after splenectomy and its primary prophylaxis with antithrombin III concentrates and danaparoid sodium in liver cirrhosis with portal hypertension. J Am Coll Surg.

[12] Wu S, Wu Z, Zhang X, Wang R, Bai J (2015). The incidence and risk factors of portal vein system thrombosis after splenectomy and pericardial devascularization. Turk J Gastroenterol.

[13] Cheng Z, Yu F, Tian J, Guo P, Li J, Chen J (2015). A comparative study of two anti-coagulation plans on the prevention of PVST after laparoscopic splenectomy and esophagogastric devascularization. J Thromb Thrombolysis.

[14] Tran T, Demyttenaere SV, Polyhronopoulos G, Séguin C, Artho GP, Kaneva P (2010). Recommended timing for surveillance ultrasonography to diagnose portal splenic vein thrombosis after laparoscopic splenectomy. Surg Endosc.

[15] Qi X, Bai M, Guo X, Fan D (2014). Pharmacologic prophylaxis of portal venous system thrombosis after splenectomy: a meta-analysis. Gastroenterol Res Pract.

[16] de Angelis N, Abdalla S, Lizzi V, Esposito F, Genova P, Roy L (2017). Incidence and predictors of portal and splenic vein thrombosis after pure laparoscopic splenectomy. Surgery.

[17] Kuroki T, Kitasato A, Tokunaga T, Takeshita H, Taniguchi K, Maeda S (2018). Predictors of portal and splenic vein thrombosis after laparoscopic splenectomy: a retrospective analysis of a single-center experience. Surg Today.

[18] Abdel-Razik A, Mousa N, Elhelaly R, Tawfik A (2015). De-novo portal vein thrombosis in liver cirrhosis: risk factors and correlation with the model for end-stage liver disease scoring system. Eur J Gastroenterol Hepatol.

[19] Ikeda M, Sekimoto M, Takiguchi S, Kubota M, Ikenaga M, Yamamoto H (2005). High incidence of thrombosis of the portal venous system after laparoscopic splenectomy: a prospective study with contrast-enhanced CT scan. Ann Surg.

[20] Tripodi A, Mannucci PM (2011). The coagulopathy of chronic liver disease. N Engl J Med.

[21] Bai DS, Xia BL, Zhang C, Ye J, Qian JJ, Jin SJ (2019). Warfarin versus aspirin prevents portal vein thrombosis after laparoscopic splenectomy and azygoportal disconnection: a randomized clinical trial. Int J Surg.

[22] Qian YY, Li K (2017). The early prevention and treatment of PVST after laparoscopic splenectomy: a prospective cohort study of 130 patients. Int J Surg.

[23] Broe PJ, Conley CL, Cameron JL (1981). Thrombosis of the portal vein following splenectomy for myeloid metaplasia. Surg Gynecol Obstet.

[24] AlSabah SA, AlRuwaished M, Almazeedi S, Al Haddad E, Chouillard E (2017). Portomesenteric vein thrombosis post-laparoscopic sleeve gastrectomy: case series and literature review. Obes Surg.

[25] Kinjo N, Kawanaka H, Akahoshi T, Tomikawa M, Yamashita N, Konishi K (2010). Risk factors for portal venous thrombosis after splenectomy in patients with cirrhosis and portal hypertension. Br J Surg.

[26] Danno K, Ikeda M, Sekimoto M, Sugimoto T, Takemasa I, Yamamoto H (2009). Diameter of splenic vein is a risk factor for portal or splenic vein thrombosis after laparoscopic splenectomy. Surgery.

[27] Lai W, Lu SC, Li GY, Li CY, Wu JS, Guo QL (2012). Anticoagulation therapy prevents portal-splenic vein thrombosis after splenectomy with gastroesophageal devascularization. World J Gastroenterol.

