# Supplementary material for: Early prevention and risk factors analysis of portal vein system thrombosis after laparoscopic splenectomy and pericardial devascularization
Source: Surg Endosc. 2022 Jun 28;36(12):8918–26. doi: 10.1007/s00464-022-09340-5 (PMC9652216; doi:10.1007/s00464-022-09340-5)
Supplement: Supplementary file 1 — Supplementary file1 (DOCX 18 kb) [file 464_2022_9340_MOESM1_ESM.docx]

| **Table S1:** The comparison of platelet level between warfarin and aspirin groups. | | | |
| --- | --- | --- | --- |
| Time point | Warfarin (n=68) | Aspirin (n=63) | *P* |
| POD 0 | 44.06±13.90 | 44.37±16.59 | 0.787 |
| POD 7 | 375.47±102.60 | 376.41±87.01 | 0.955 |
| POD 14 | 310.04±95.65 | 302.92±80.80 | 0.647 |
| POM 1 | 247.44±65.90 | 242.54±72.69 | 0.686 |
| POM 3 | 240.79±72.95 | 235.10±85.58 | 0.611 |
| POM 6 | 205.44±65.30 | 217.97±69.95 | 0.169 |
| POM 12 | 198.84±72.94 | 201.57±64.45 | 0.520 |
| Data shown as mean ± standard deviation, as indicated.  POD, postoperative day; POM, postoperative month. | | | |
