# Supplementary material for: Early prevention and risk factors analysis of portal vein system thrombosis after laparoscopic splenectomy and pericardial devascularization
Source: Surg Endosc. 2022 Jun 28;36(12):8918–26. doi: 10.1007/s00464-022-09340-5 (PMC9652216; doi:10.1007/s00464-022-09340-5)
Supplement: Supplementary file 2 — Supplementary file2 (DOCX 18 kb) [file 464_2022_9340_MOESM2_ESM.docx]

| **Table S2:** The comparison of platelet level between non-PVST and PVST groups. | | | |
| --- | --- | --- | --- |
| Time point | Non-PVST (n=83) | PVST (n=48) | *P* |
| POD 0 | 43.60±13.97 | 45.25±17.21 | 0.871 |
| POD 7 | 372.64±88.75 | 381.60±105.84 | 0.605 |
| POD 14 | 301.30±98.37 | 315.81±68.35 | 0.368 |
| POM 1 | 240.43±66.27 | 253.13±73.56 | 0.312 |
| POM 3 | 236.78±81.15 | 240.25±75.99 | 0.715 |
| POM 6 | 215.63±66.71 | 204.27±69.24 | 0.238 |
| POM 12 | 195.63±62.31 | 207.98±78.72 | 0.394 |
| Data shown as mean ± standard deviation, as indicated.  POD, postoperative day; POM, postoperative month. | | | |
